# How voluntary orienting of attention and alerting modulate costs of conflict processing

**DOI:** 10.1038/srep46701

**Published:** 2017-04-24

**Authors:** Alberto Zani, Alice Mado Proverbio

**Affiliations:** 1 Institute of Molecular Bioimaging and Physiology (IBFM), National Research Council (CNR), Milan, Italy; 2 Milan-Mi Center for Neuroscience, University of Milano-Bicocca, Milan, Italy; 3 Department of Psychology, University of Milano-Bicocca, Milan, Italy

## Abstract

There is evidence that pre-cued valid orienting of attention to competing information diminishes costs of brain processing of conflict. Still unclear, because scantily addressed by neuroimaging studies and mostly analyzed by means of behavioral indexing, it is whether conflict undergoes an equivalent modulation by tonic and pre-cued phasic alerting. Here, we investigated the functional relationships between attention orienting, alerting and executive systems using the Attention Network Test (ANT). Both reaction times (RTs) and ERPs were recorded. In line with previous literature, results showed that both RTs and a so-called ERPs conflict negativity (*CN*), prominent at anterior scalp and indexing conflict processing, were positively modulated by a prior valid orienting of attention onto the location of conflicting stimuli. Indeed, in this condition both kinds of markers showed faster latencies, while CN also reached higher amplitude values than in both alerting conditions, and, in turn, in pre-cued phasic alerting than in uncued tonic alerting. Moreover, while CN was larger over the right hemisphere independent of functional conditions, it was strongly modulated by the latter over the left hemisphere. Our ERP findings support the views of conflict modulation by both orienting and phasic alerting and of a functional integration between attentional brain networks.

At present, a bulk of evidence has accumulated by means of both behavioral and neurofunctional studies that the attention system of the human brain consists of three main attention networks: namely, an *alerting* system, deputed to the achievement and maintenance of an adaptive alert state, a *spatial orienting* system, involved in the selection of information from relevant locations of the visual space, and an *executive control* system, which detects, monitors and resolves conflicting aspects within the information processing stream. Originally, the theorized distinction among these three networks also held the universally agreed assumption of an almost complete functional independence between brain networks for executive control and those for covert visual spatial attention orienting and alerting^1^,^2^,^3^. With the goal to provide support for this theoretical view and to investigate the functional dynamics and efficiency of the three separate networks of attention, as well their influences on behavioral overt responses, Fan *et al*.^2^ devised a computerized Attention Network Test (or ANT) and its relative computational scoring methods. These computational methods tapped at the executive network based on the universally accepted tenet that the latter network was unaffected by the different alerting and/or orienting attention conditions^2^,^3^. Using the aforementioned procedural scoring method, initially the theoretical view of the networks independence received strong support^1^,^2^,^3^. Rueda *et al*.^4^ further supported this same view measuring RTs in association with the ANT paradigm, despite computing separate conflict scores as a function of each attentional cueing condition. In fact, these authors observed the development of the attentional networks in a cross-sectional experiment where behavioral performance was observed with four age groups ranging from 6 through 9, 10 years, and adults. Hence, they concluded that while alertness showed evidence of change up to and beyond age 10, conflict scores appeared to be stable after age seven and orienting scores did not change across attentional precueing conditions in the age ranges studied.

Despite this initial support, the network independence assumption was challenged by several later studies on the relationships between attentional preparation and filtering processing of conflict-inducing irrelevant distracters, as based on behavioural and ERPs as well as fMRI methods. Notwithstanding the wide differences in the somehow distinct processes measured by these studies, all in all, their findings imply that (1) preparatory brain activity accompanying shifts of covert attention and conflict resolution tend to exhibit a large degree of overlap and integration, and (2) preparatory processes are modulated by the foreknowledge of visual information competition during covert attention (e.g., see refs 5, 6 and 7). For truth sake, however, it has to be indicated that, based on behavioral evidence (i.e., RTs), the other-way around view has been advanced that it is conflict processing to be positively modulated by a previous valid orienting of covert attention to the location of following conflicting target stimuli, and negatively modulated by the previous manifestation of a mere phasic alerting reaction by Fan *et al*.^8^. Indeed, in this study, using a revised version of ANT and its scoring procedures, these authors found that, whereas alerting improves overall response speed, it exerts a negative influence on executive control under certain conditions. Moreover, the authors reported that a valid orienting cue can enhance but an invalid cue can diminish the capacity of executive control to overcome conflict. More recently, Spagna *et al*.^9^ investigated the hemispheric contributions to the attentional networks using two different lateralized readapted versions of ANT. One version, presented the original arrow-arrays while the other version had fruits-arrays as target and flankers. Data collected from forty-seven participants showed a left visual field advantage for invalid unattended targets.

Although important for investigating the influences of activation and/or of a lack of activation of the attentional networks on behavioral responses and coping cognitive strategies, unfortunately behavioral studies^10^,^11^,^12^,^13^,^14^,^15^ cannot tell much about the timing, localization and activation dynamics of brain underpinnings of the conflict resolution mechanisms and about the kind of influences of valid spatial orienting on its resolution.

In these regards, both electrophysiological and hemodynamic neuroimaging revealed much useful for providing new insights on this matter. Nonetheless, the bulk of studies based on the measurement of both type of the aforesaid brain signals mostly pursued the investigation of error- and conflict-related decisional processes, independently of the influence of the alerting and/or orienting attention conditions.

Indeed, early ERP studies dealt with these matters focusing their heed on late-latency negativities recorded at the anterior sites of scalp surface after the delivery of an informational conflicting stimulus target. More specifically, they directed their attention on the late-latency N200 component found to be directly related to executive processing. Indeed, N200 was found to be elicited by stimuli associated with initial response inhibition in paradigms like the flankers task and was thought to reflect response conflict in correctly responded trials^16^,^17^,^18^. Interestingly, the anterior cingulate cortex (ACC) has been suggested as a neural generator for this scalp-recorded N200^17^.

Unlike these original studies, more recent investigations focused their analyses of the aforementioned negativities on a so called *conflict negativity (CN*), proposed to be a neurophysiologic reflection at the scalp of decisional conflict during personal decision-making^19^. This response consists in a negative deflection of ERPs that appears within 60 ms of making a behavioral response based on a conflict-related decisional process^20^,^21^,^22^, and the harder is the decision between two conflicting information, the larger is the CN response^23^,^24^,^25^. Importantly, *CN* too is believed to be generated by a conflict-monitoring system localized in the ACC. Literally, neuroimaging studies (e.g., Botvinick *et al*.^26^ have shown the crucial role of ACC in monitoring and resolving perceptual and response conflicts in this contrasting tasks. Most interestingly, in an original ERP experiment Yeung *et al*.^27^ confirmed predictions of the connectionist conflict monitoring model indicating that, unlike on error trials, where the response conflict is following the motor response, as mirrored at the scalp surface by an error-related-negativity (or ERN), when conflict occurs on correct trials, it is seen almost exclusively prior to the motor response, as indexed by the N2 component of ERPs, occurring in an earlier-latency range than that of the ERN. A direct implication of this model is that, while both components are localized to ACC, their differing latency of appearance should depend by a difference in the timing of activation of the latter conflict monitoring area of the brain.

More recently, the combined fMRI and EEG study by Siemann *et al*.^28^ have provided evidence that the ACC is more involved in solving early top-down conflicts with invalid trials (reflected on scalp surface by a frontocentral negativity). Most importantly, a study by Iannaccone *et al*.^29^ was able to spatially and temporally dissociate conflict and error processing using simultaneously recorded EEG and fMRI data during a modified Flanker task. This study found that ACC activation was more related to error processing while conflict monitoring was related to an activation of Supplementary Motor Area (SMA).

Much interestingly, the studies which investigated the relationships between the covert orienting of attention and the attention alerting conditions with target-related conflicting processing have only separately addressed and partly solved these issues. For example, the more recent ERP studies dealing with covert attention, but not with alerting, advanced that brain activation patterns related to orienting of covert attention and conflict resolution were connected, in that attentional preparatory processes were modulated by the foreknowledge of visual information conflicting features (e.g., see ref. 6, 7, and 8).

Despite the relevant evidence of a functional interrelationship between neural networks regulating top-down anticipatory attentional guidance and executive suppression of irrelevant visual distracters provided by the reviewed studies, at present there is still an indisputable lack of knowledge of the possible differences in the timing and features of monitoring and suppressing mechanisms as a function of pre-target voluntary - knowledge-driven - preparatory attentional orienting and/or post-target reflexive - data-driven - orienting, and, most importantly, of more basic, pre-target phasic and/or post-target tonic alerting states.

Purpose of the present study was to investigate whether neurofunctional processing of conflict-inducing information, as reflected by both RTs and scalp-recorded ERPs, might be differently modulated by visual attention covert orienting and/or alerting. More specifically, we sought to determine whether brain executive control processing of conflicting information during a *pre-cued* voluntary orienting of spatial attention might have a different time course and neural undergirding in comparisons to those same processes during a *post-target* reflexive orienting of attention. Again, we also sought to determine whether, tout-court, phasic alerting states differently modulated conflict processing from task-related tonic alerting states. Last but not least, we also tapped at possible differences in hemispheric lateralization of the aforementioned neurofunctional mechanisms.

## Material and Methods

### Participants

Thirteen right handed students (8 women and 5 men) ranging in age between 20 and 30 years (mean age = 23.4; SD = 1.98) took part in the study. All had a normal or correct to normal vision and reported no history of neurological illness or drug abuse. Before to be enrolled in the study, the students were administered an Italian version of the Edinburgh Handedness Inventory, which is a paper-and-pencil Likert-scale-type questionnaire for self-assessment of hand-dexterity and ocular laterality preference. This scale indicated strong right-handedness and right ocular dominance in all participants. Experiments were conducted with the understanding and written consent of each participant according to the Declaration of Helsinki (BMJ 1991, 302:1194) with approval from the Ethical Committee of the National Research Council and in compliance with APA ethical standards for the treatment of human volunteers (1992, American Psychological Association). None of the participants was paid, but all obtained academic credits (i.e., CFU) for their participation in the study.

### Stimuli

**Figure 1:**
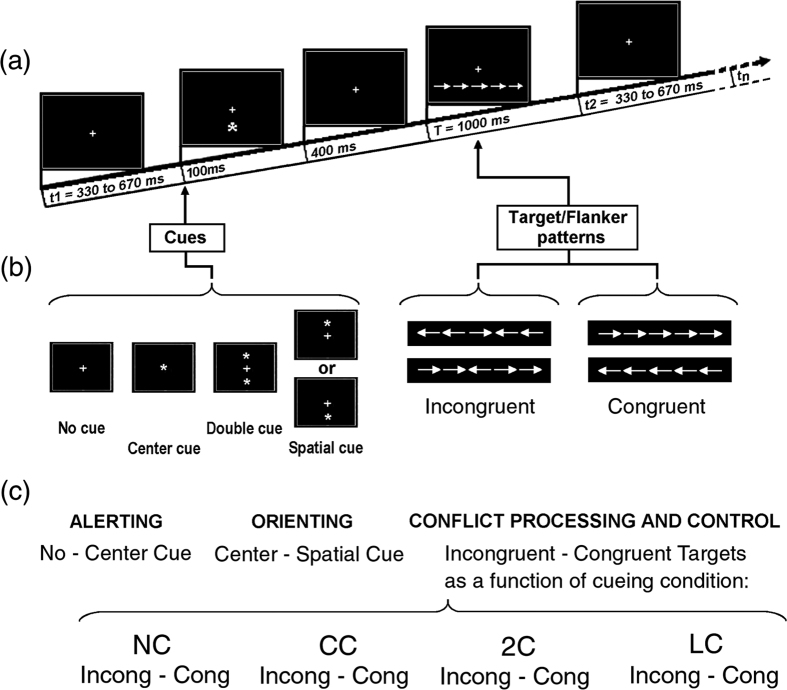
Schematic representation of the cue-target stimulation procedures used in our study. (**a**) Representative timeline of experimental stimulation for each trial presented. Overall, a trial lasted 1500 ms and the inter-trial interval (ITI) randomly varied between 330 and 670 ms. (**b**, Right) Target stimuli: the four possible combinations of central arrow-targets with lateral arrow-flankers are shown here. Leftward- or rightward-pointing arrow-targets with directional congruent arrow-flankers distracters are presented on the right; on the left, instead, targets with directional incongruent flankers (distracters) are drawn. (**b**, Left) Cue types used in the four experimental conditions: NC = No Cue; CC = Center Cue; 2 C = Double cue; LC = Local Cue validly informative of target spatial location. More specifically, for LC condition the two possible spatial positions for the appearance of spatial cues above or below the fixation point are drawn (Drawn and modified from Neuhaus *et al*.^30^. (**c**) Scoring procedures for calculating attention network functions. Conflict effects were derived by means of the subtraction of the average response to congruent targets from that to incongruent targets separately for each ANT cueing condition.

An adapted version of original cue-target ANT^2^, combining a cued attentional task with the flanker task, as illustrated in Figure 1a and b based on Neuhaus’s *et al*.^30^ readapted depiction, was used in this study. Target-flankers conjoined stimuli consisted in five horizontally disposed, contiguous, white-colored arrows, of which the central one, pointing rightwards or leftwards, would be task-relevant (indicating the correct spatial side for a correct RTs-recording hand button-press), while the lateral ones (or flankers) would act as potential distracters. Flankers’ arrowhead pointed either toward the same direction as the leftward or rightward true-target arrowhead (so called *congruent flankers*) or in the opposite direction of the true-target arrowhead (so called *incongruent flankers*). This way, four different types of target-patterns, eliciting different levels of perceptual and response decisional conflict, were created: rightward target- rightward congruent flanker patterns and leftward target-leftward congruent flanker patterns, as well as rightward target-leftward incongruent flanker patterns and leftward target-rightward flankers incongruent patterns (see Fig. 1b, right side).

Volunteers faced a 17” personal computer (PC) screen placed at 114 cmfrom their eyes. The PC stimulus background was black and a white fixation point (a cross subtending 0.25 degrees of visual angle) was always present at the screen centre. The four white arrow-patterns were always presentedin random order above or below the fixation point at a vertical distance of 1.25° degrees of visual angle from it, as referred to the screen vertical meridian. Overall, as referred to the screen horizontal meridian, they subtended 9° of visual angle, 4.5° of which fell to the left and 4.5° to the right of the screen vertical meridian, the central true target-arrow of the patternfalling perfectly centered on the latter meridian (see Fig. 1a again).

The administration of these four target configurations could be preceded, or not, by the presentation, 500 ms prior of the target, of different stimulus patterns, thus creating four different pre-cueing attentional conditions (see Fig. 1b, left side), namely:

#### No Cue (NC)

No cue was presented before arrow-targets patterns. This way, neither warning nor spatially centered information was provided to the volunteers before targets administration.

#### Center Cue (CC)

500 ms prior target presentation, an asterisk, subtending 0.25°, appeared for 100 ms at the centre of the screen, overimposed on the fixation cross. Thus, albeit purposefully pre-warning of the target delivery, this pre-cueing mode was, however, not informative of the location at which the target stimulus would have been later on presented above or below the fixation cross.

#### Double *Cue* (2C)

500 ms prior target presentation, two asterisks (each subtending 0.25°) appeared simultaneously for 100 ms, below and above the fixation point. Overall, the two asterisks subtended a spatial spotlight width of 2.5°, centered on the vertical meridian. Although being informative of the following target delivery, as for the above CC condition, this pre-cueing mode too did not provide any information about whether the target stimulus would be presented above or below the fixation cross.

#### Local Cue (LC)

500 ms before target presentation, a single asterisk, subtending 0.25°, appeared for 100 ms of duration above or below the fixation point, centered at 1.25° of eccentricity along the screen vertical meridian (see Fig. 1a and b). This vertically-lateralized, single cue provided validly predictive information of both the delivery of a target and of its location before the target was presented at that same location.

### Procedure

The experiment was conducted at the Cognitive Electrofunctional Imaging Lab of the Institute of Molecular Bioimaging and Physiology, of National Research Council (Milan, Italy). InstEP software package (InstEP Inc., Ottawa, Canada), run on a local network made up of two personal computers (PCs), was used for stimulus presentation and EEG data recording as well as for offline analyses.

For each of the four cueing conditions 45 congruent and 45 incongruent arrow-targets were presented as target (45 above and 45 below the fixation point) for a total number of 360 stimuli. Stimulus presentation was sequenced in 12 trial blocks, including 3 blocks for each attention condition (2C, CC, NC, LC) intermixed by short pauses of a few minutes. The order of blocks presentation was counterbalanced across participants. Each trial block began with three warning signals - “Set”, “Ready”, and “Go” – sequentially flashed with an ISI of 250 ms, inviting the participant to concentrate and get ready for the run about to begin.

Except for the NC condition, on each trial of the 3 pre-cued attention conditions (i.e., CC, 2C, LC), a different cue-type was presented for 100 ms. Then, after a fixed ISI of 400 ms, one of the four congruent or incongruent target-flanker patterns was presented above or below the fixation cross for 1 sec (i.e., from 500 to 1500 ms post-cue presentation). Intertrial interval (ITI) was 150 ms to which a random time span ranging from 330–670 ms was added, overall leading to a random ITI of 1830–2170 ms (see Fig. 1a). While for the CC and 2C conditions, the cue-target spatial locations were totally randomly associated, in the LC condition the above association was pseudo-random, in that an upper or lower single asterisk was always validly followed by one of the target-patterns at that location, independently of the target leftward-rightward pointing direction and of flankers directional congruency. During active recording, participants were instructed to stare at a screen central fixation cross, to not move and to avoid both horizontal and vertical eye movements and blinks. The task consisted in responding on the basis of the pointing direction of the central arrow (i.e., leftward or rightward), while ignoring all the other information, by pressing a button as quickly and accurately as possible with the laterally corresponding left or right index finger. To familiarize participants with the tasks, before starting the experiment, some short practice runs were administered.

### EEG recordings and analysis

Discreet electroencephalogram (EEG) sweeps were recorded from scalp electrodes mounted in a 64-electrode ECI elastic electro-cap. Only 30-electrode scalp-sites were used. The electrodes were located at frontal (Fp1, Fp2, Fz, F3, F4, F7, F8), central (Cz, C3, C4), temporal (T3, T4), posterior temporal (T5, T6), parietal (PZ, P3, P4) and occipital (O1, O2) scalp sites of the 10–20 System devised by Jasper (1958) for the International EEG Federation. Additional electrodes based on the later 10–10 System were placed at an anterior frontal site (AFz), halfway between frontal and central sites (FC1, FC2, FC5, FC6), central and parietal sites (CP5, CP6), parietal and occipital sites (PO3, PO4) and posterior temporal and occipital sites (OL/PO7, OR/PO8). Vertical eye movements were recorded by two electrodes placed below and above the right eye, and horizontal eye movements were recorded by electrodes placed at the outer canthi of the eyes. Linked-earlobes served as the reference lead, whereas an electrode included in the cap between Fp1 and Fp2 but 0.6 in. (1.5 cm) below them was used as a ground site. The EEGs and EOGs were amplified with a half-amplitude band pass of 0.16–50 or 0.02–50 Hz, respectively. Amplifier gain for the EOG was 0.5 times that for EEG. Electrode impedance was kept below 5 kΩ. Event-related potential (ERP) sweeps went from 100 ms before (−100 ms) to 1500 ms after cue presentation. Target appearance (i.e., onset) on the PC-screen started 500 ms after cue presentation and lasted for the remaining 1000 ms of the EEG sweeps, so to avoid any possible target-disappearance-related (i.e., offset) EEG response. Discreet EEGs and EOGs sweeps were digitized at a rate of 512 Hz. The preparation and experiment lasted almost 2 h overall. Offline, automated rejection of electrical artifacts was performed before EEG averaging for any participants to discard epochs in which eye movements, blinks or excessive muscle potentials occurred. The artifact rejection criterion was a peak-to-peak amplitude exceeding ±90 μV for EEG signal or ±120 μV for EOG signal, and the rejection rate was ∼5%. ERP epochs associated with behaviorally incorrect or delayed (i.e., falling after 1500 ms) responses were also rejected.

Unlike ANT^2^ original scoring procedures determining behavioral conflict cost effects by means of the subtraction of the mean RTs to all congruent flanking targets from the mean RTs to all incongruent targets, as collapsed across all pre-cueing conditions, which have also been used for investigating the neurofunctional and neuroanatomical undergirdings of conflict processing by means of both ERPs and fMRI (e.g., see ref. 3), we conformed to the modified scoring procedures proposed in a later behavioral study by Fan’s *et al*.^8^. Hence, in accordance with these procedural standards, we computed average ERPs to both incongruent and congruent target-types, as well as the difference-waves (DWs) between the above mentioned ERPs, separately for the 4 cueing conditions. ERP signals were then grand-averaged across the participants’ group. However, the data of 2 participants had to be excluded from grand-averaging and further analyses for excessive EEG and EOG artifacts.

**Figure 2:**
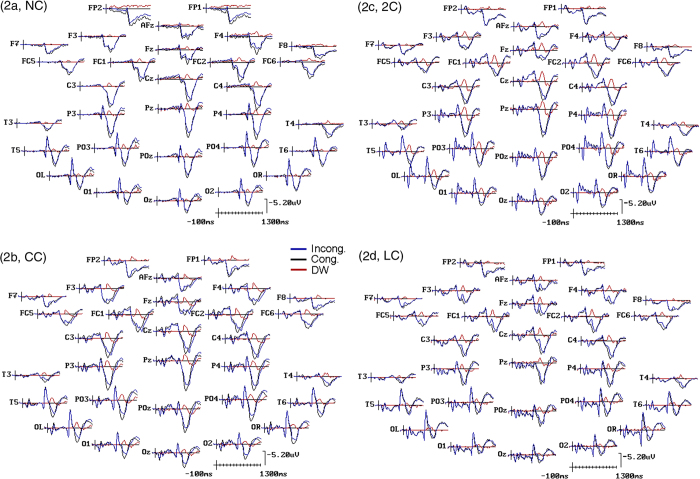
Grand-average ERPs to incongruent and congruent target-flankers patterns obtained at the 30 scalp electrode sites as a function of the four pre-cueing conditions. Overimposed onto the grand-averaged ERPs, the DWs computed subtracting ERPs to the congruent targets from ERPs to the incongruent ones, are also reported. It is noteworthy that in the DWs for all cueing conditions, namely, NC = No Cue (**a**), CC = Center Cue (**b**), 2 C = Double cue (**c**) and LC = Local Cue validly informative of target spatial location (**d**), a prominent post-target (i.e., 150–460 ms, that is, 650–960 ms post-cue) negativity, defined as *conflict negativity (CN*), was obtained. This activation showed to be larger at anterior pre-frontal than posterior scalp sites, and medially than laterally, independent of attentional pre-cueing. Note also that both ERPs and DWs have been plotted with an expanded time scale going from −100 ms pre-cue until 1300 ms post-cue to highlight any possible *CN* morphological differences across the cueing conditions all over the scalp.

**Figure 3:**
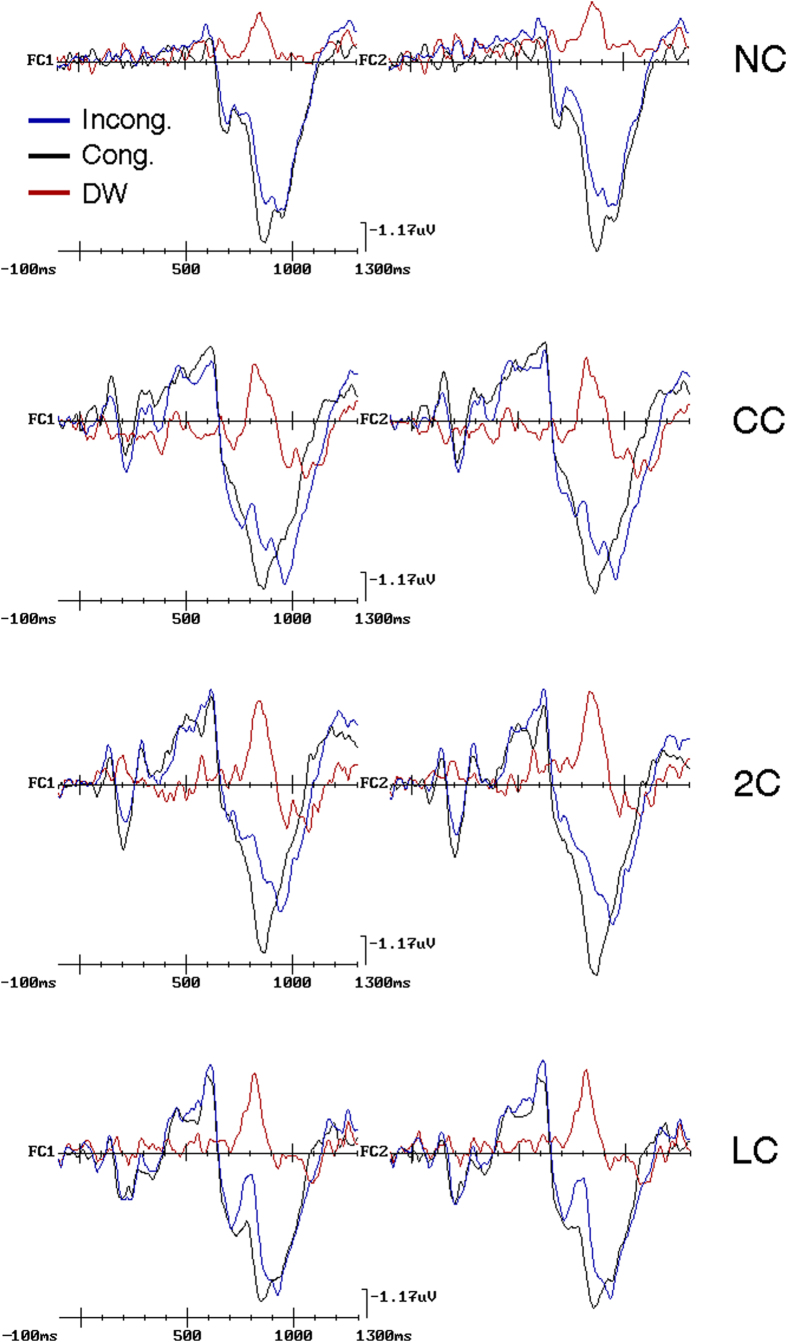
Grand-average ERPs to incongruent and congruent target-flankers patterns obtained at FC1 and FC2 homologous scalp electrode sites as a function of the four pre-cueing conditions: NC = No Cue; CC = Center Cue; 2C = Double cue; LC = Local Cue validly informative of target spatial location. Overimposed onto the grand-averaged ERPs, the conflict-related DWs computed subtracting ERPs to the congruent targets from ERPs to the incongruent ones, are also drawn. As anticipated in Fig. 2, in the DWs for all cueing conditions a clear-cut post-target *conflict negativity (CN*) was obtained for all cueing conditions. Note that as for Fig. 2, ERP wave forms have been drawn with an expanded time scale to highlight *CN* morphological features.

Grand-averaged ERPs to incongruent and congruent target-flanker combinations as a function of the four different cueing conditions, together with their difference obtained by subtracting ERPs to congruent target trials from ERPs to incongruent target trials, are illustrated in Fig. 2. The DWs thus obtained revealed prominent post-target negativities in all cueing conditions, which in line with the recent neurofunctional literature on conflict monitoring and resolution reviewed above, we addressed as a *conflict negativity (CN*). Although appearing of different height and latency as a function of cueing conditions, these negativities showed to be overall of greater height at anterior medio-lateral precentral and frontocentral scalp electrodes, and degraded at more lateral and posterior sites of the scalp in all conditions (see Fig. 2 again). In order to gain a better angle of the relationships of the *CN* with N2, P3a and P3b components, Fig. 3 plots selective grand-average ERPs recorded in response to the different types of congruent and incongruent flankers at a most representative homologous pair of fronto-central electrodes, where post-target negativities were clearly more prominent, namely FC1 and FC2, as a function of cueing types. On the one hand, although with different amplitudes and latencies across cueing conditions, an enhancement of N2 is evident for incongruent targets no matter the condition taken into account. On the other hand, although with differences across cueing conditions, a lower and later P3a and P3b complex was also evident in response to the incongruent trials, with respect to the congruent ones, in all these conditions. Overall, then, as a consequence of the mentioned latency jitter and amplitude changes across conditions, *CN* amplitude and latency in the DWs overimposed on raw waveforms seem to correspond closely to the differences in N2 amplitude and latency between incongruent and congruent target-flanker patterns mostly when attention was voluntarily focused onto the targets location prior of their delivery, that is in the LC condition. Conversely, in the remaining cueing conditions, besides to N2, *CN* gave the impression to be consistently related in increasing degrees to later latency differences in P3 complex responses between the different levels of targets congruency.

**Figure 4:**
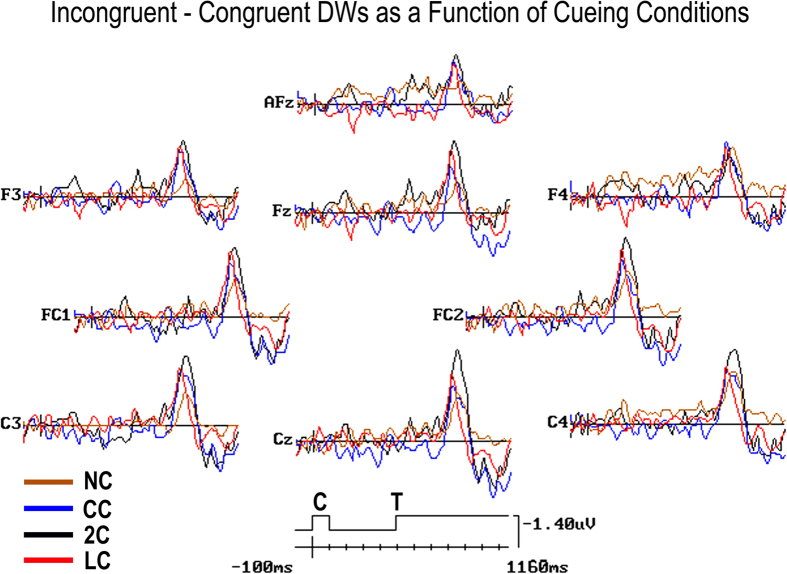
Grand-averaged ERP-DWs *CN* from midline (i.e., AFz, Fz, and Cz), and lateralized F3, F4 (frontal), FC1, FC2 (Fronto-central), and C3, C4 (central) electrode sites as a function of the four cueing conditions: NC = No Cue; CC = Center Cue; 2C = Double cue; LC = Local Cue validly informative of target spatial location. DWs have been drawn with an expanded time scale going from −100 ms pre-cue to 1160 ms post-cue to further highlight post-target *CN* morphological peak latency and amplitude differences and similarities at these anterior scalp sites across pre-cueing conditions in between 650–960 ms after cue types delivery (i.e., 150–460 ms after target delivery). C = Cue appearance time and duration; T = Target appearance time and persistent duration on the screen.

To asses these empirical eyeball observations, amplitude and latency values of *CN* peak in the ERP-DWs for each participant were then automatically detected and measured by InstEP software package as a function of each cueing type, with respect to the average baseline voltage at sites where it reached its maximum greatness: namely, at frontal (F3, F4), fronto-central (FC1, FC2), and central (C3, C4) homologous sites as, in general, suggested by Fig. 2. Latency range for measurements was set up in between post-target 200–500 ms (i.e., a post-cue present/absent latency of 700–1000 ms), based on *CN* time progression observed in Fig. 3, and, more directly, in Fig. 4, illustrating *CN* obtained at all anterior scalp leads as a function of cueing conditions, overimposed one another. Voltage distribution maps^31^,^32^,^36^ of scalp-recorded ERPs were also computed to explore more thoroughly the influence of the different cueing conditions on the *CN* scalp distribution and temporal course, besides to obtain somehow evidence of an involvement of the ACC in the *CN* generation.

As for electrophysiological measures, we followed the same operational mode for determining behavioral conflict-related effects for RT speed and accuracy measures. Overall, we carried out the aforementioned measurements and computations separately for any of the cue types, in order to explore the influence of pre-target phasic alerting and/or voluntary top-down attention orienting as well as post-target alerting and/or reflexive attention orienting on detection and resolution of information conflict.

### Statistical analyses

Behavioral data, namely both motor response accuracy and speed, underwent two separate repeated-measures 2-ways ANOVAs whose factors of variability were: task (4 levels) and congruence (2 levels).

Two three ways repeated-measures ANOVAs were performed on mean latency and amplitude values of CN response. Factors of variability were: cueing condition (4 levels: NC, CC, DC, LC), electrode (frontal, fronto-central, and central), and hemisphere (left, right). Together with the repeated-measures ANOVA we reported generalized effect sizes^37^,^38^, η^2^_g_, for both the behavioral and electrophysiological analyses. The Greenhouse-Geisser correction was also applied to compensate for possible violations of the sphericity assumption associated with factors which had more than two levels. In this case, the degrees of freedom accordingly modified are reported together with the epsilon (ε) and the corrected probability level. Post-hoc comparisons among means for significant factors with more than 2 levels were performed by means of Tukey and Duncan tests.

Last but not least, challenged by reviewers’ comments, to deal with ANOVAs between-subject variability amount of uncertainty as related to within-subject effects in repeated-measurement designs, we applied Denis Cousineau’s (2005)^34^ alternative solution to the Loftus and Masson’s (1994)^33^ computation of confidence intervals or error bars for dealing with this possibly misleading problem, as well as the simple correction of Cousineau’s (2005) confidence intervals by means of Morey’s^35^ method.

## Results

### Behavioural results

**Figure 5:**
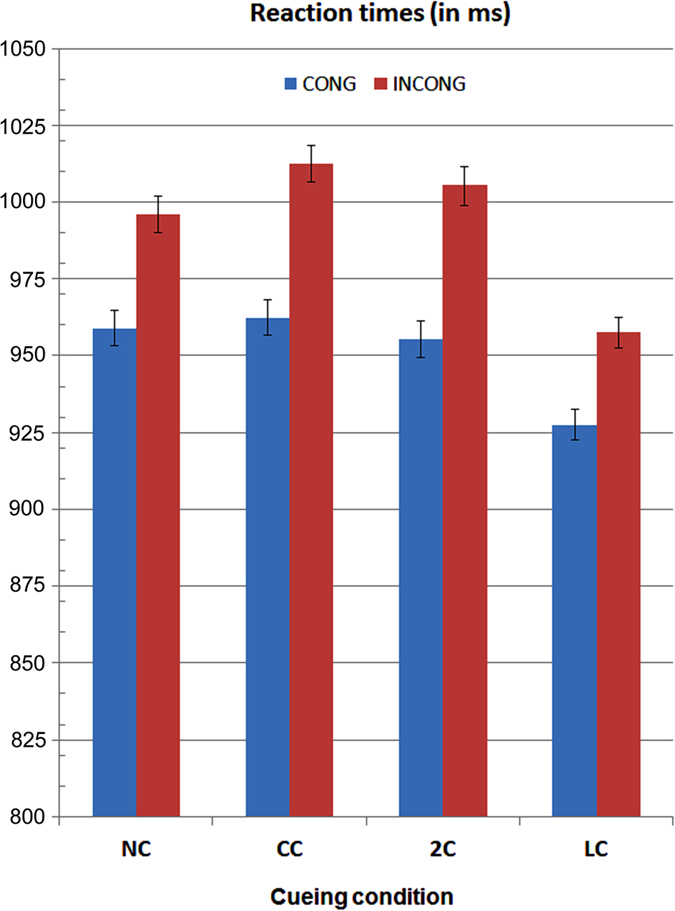
Mean reaction times and standard errors (S. E. - in ms) to targets computed as a function of the four cueing conditions, namely NC = No Cue; CC = Center Cue; 2C = Double cue; LC = Local Cue validly informative of target spatial location, and target-flankers’ congruence. Note that, for a better comparison with electrophysiological indexes latency values, a time span of 500 ms is added to the volunteers’ motor responses to targets, as related to cue appearance or omission prior target delivery.

The ANOVA performed on mean RTs yielded the significance of pre-cueing-type factor (F3,30 = 9.97; p < 0.0001, ε = 0.80; η^2^ = 0.57). Post-hoc comparisons showed that response times were much faster (p < 0.01) in the LC (446 ms) than in all other conditions (2C = 476 ms; CC = 479 ms; NC = 472 ms). The ANOVA also yielded the significance of congruence factor (F1,10 = 56.84; p < 0.000001, ε = 1; η^2^ = 0.83). RTs were much faster to congruent (449 ms) than incongruent (488 ms) targets. The further interaction of task x congruence (F3,30 = 3.673, p < 0.023, ε = 0,51; η^2^ = 0.35) and relative post-hoc comparisons indicated that, while RTs to incongruent were slower than RTs to congruent targets for all cueing conditions, pre-target valid attentional orienting in LC condition notably reduced costs for incongruent vs. congruent targets processing, in that their difference did not differ significantly (see Fig. 5 for mean RTs). Furthermore, overall differences among cueing conditions were more pronounced for the incongruent than congruent targets.

As for error percentages, they did not significantly differ as a function of cueing type (i.e., 2C = 2.25%, CC = 3.08%, LC = 2.25%), but tended to increase for incongruent (3.675%) than congruent (1.085%) trials.

### Electrophysiological results

In this study we compared the electrophysiological activity recorded after target appearance to observe the effects of pre-cueing conditions (2C, CC, LC, NC) on the ability to filter the conflict induced by flanker stimuli. It was observed the presence of a large “*conflict negativity*” (CN) with a dorsolateral prefrontal and precentral maximum at a post-target latency of about 200–500 ms in all cueing conditions, as can be visible in Fig. 3. As expected, this CN response showed both latency and amplitude differences and similarities as a function of pre-cueing conditions, as confirmed by ANOVAs.

#### CN peak latency

**Figure 6:**
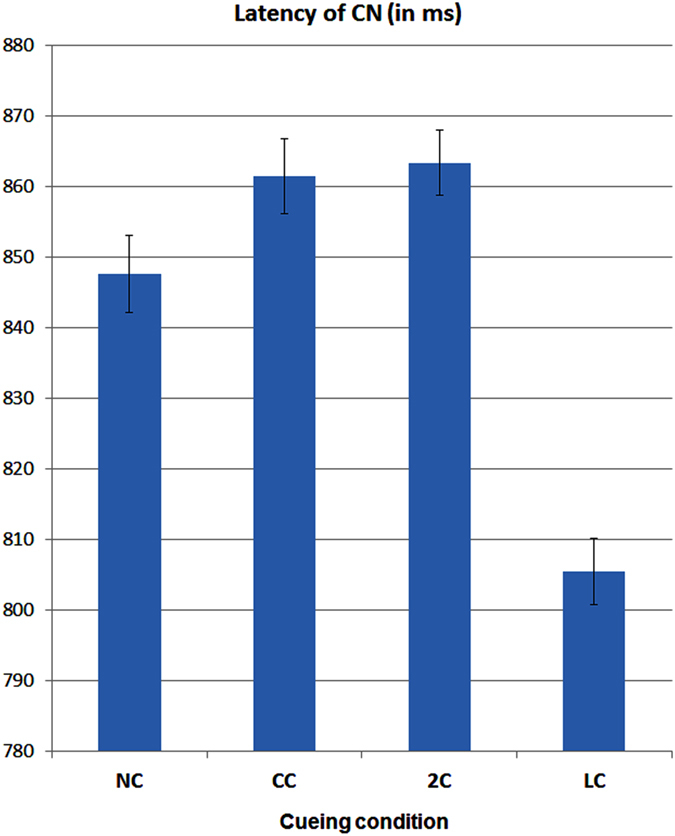
Mean peak latency values ad standard errors (S. E.) of target-related *CN* as obtained from the differences between the incongruent and congruent flankers conditions as a function of pre-cueing types, namely, NC = No Cue; CC = Center Cue; 2C = Double cue; LC = Local Cue validly informative of target spatial location. Note that the values were computed starting from cue appearance, that is, 500 ms before target delivery.

**Figure 7:**
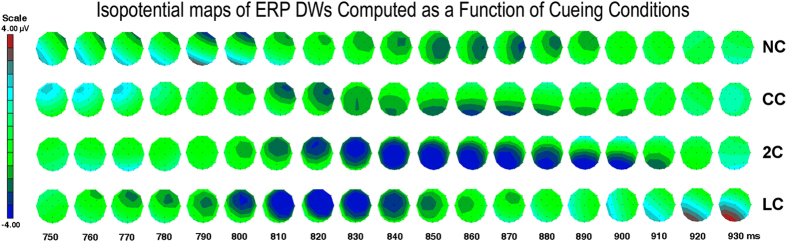
Timeline of rainbow-colors-coded scalp-recorded isopotentials maps computed on the difference-waves obtained by subtracting ERPs to congruent from those to incongruent targets in between 250–430 ms after their appearance (i.e., from 750 to 930 ms post-cue delivery or omission), with 10 ms steps, as a function of each pre-cued attention condition: NC = No Cue; CC = Center Cue; 2C = Double cue; LC = Local Cue validly informative of target spatial location. Note that a larger *CN* shows earliest starting and fading latencies in response to the conflicting information when the spotlight of spatial attention has been already oriented to its location prior than the occurrence of the latter information, namely, for LC with respect to all the other cueing conditions.

The ANOVA performed on CN peak latency yielded the significance of cue type factor [F2.65,26.48 = 7.45, p < 0,001; ε = 0,883; η^2^ = 0.44]. Post-hoc comparisons showed much earlier peak latency during LC than all other conditions (see means and S. E. values in Fig. 6). The ANOVA also yielded the significance of hemisphere factor [F1,10 = 8.9, p < 0,015; ε = 0,84; η^2^ = 0.47] with earlier CN latencies recorded over the right (840,79 ms, S. E. =5.99) than left hemisphere (848,06 ms, S. E. =4.8). The prioritized processing of target-related conflict during LC pre-cued valid orienting of attention condition is also strongly supported by the temporal series of isopotential maps of *CN* obtained by subtracting ERPs to congruent targets by ERPs to incongruent targets in between 750 to 930 (with a 10 ms step) for each attention condition, visible in Fig. 7.

Indeed, as confirmed by *CN* peak latency analysis, the advantage for target stimuli preceded by LC was of about 50 ms, with an onset at 760 ms after this spatially informative *cue* (i.e., about 260 ms after target presentation) at Fz site. Besides this much earlier onset of anterior negativity for LC than all other conditions, as can be appreciated in Fig. 6, conspicuous highness effects, with larger *CN* responses for LC and 2C conditions, were found to be significant by ANOVA (see Fig. 4 once again).

#### CN peak amplitude

**Figure 8:**
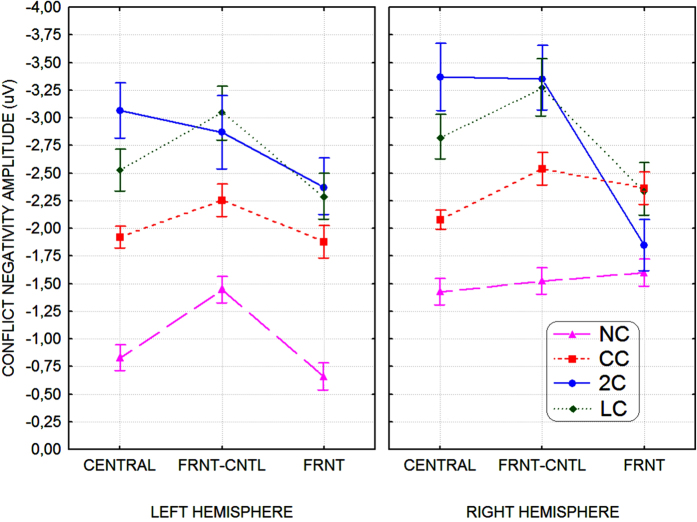
Mean amplitude values of target-related *CN* obtained at central (CENTRAL), fronto-central (FRNT-CNTL), and frontal (FRNT) recording sites of the left and right brain hemispheres as a function of the pre-cueing conditions: NC = No Cue; CC = Center Cue; 2C = Double cue; LC = Local Cue validly informative of target spatial location. Note that for any within-subject level values, confidence interval (CIs) bars not including variability associated with between-subject differences are reported. CIs were computed by means of Cousineau’s (2005) and Morey’s (2008) methods.

The ANOVA performed on *CN* amplitude yielded the significant effect of electrode [F_2,19.98_ = 5, p < 0,017; ε = 0,999; η^2^ = 0.50] and the interaction of cueing condition x electrode x hemisphere [F_4.99,49.9_ = 2.55, p < 0.039; ε = 0,813; η^2^ = 0.22]. Relative post-hoc comparisons showed that *CN* was larger at frontocentral (−2.53, S. E. = 0.64 μV) than other sites (central = −2.25, S. E. = 0.52 μV; frontal = −1.91, S. E. = 0.51 μV; see Fig. 8). The triple interaction showed that, overall, CN was larger over the RH than LH (p < 0.001), especially when no cue was presented (NC condition). Post-hoc comparisons also revealed that *CN* response to targets was much larger in the 2C and LC conditions than in the CC condition (p < 0.001 for both comparisons), thus indicating an effect of pre-cued valid spatial orienting of attention, and, in turn, in the CC than NC conditions (p < 0.001), thus also hinting at an effect of previous alerting. *CN* was modulated by cueing condition, especially over the left hemisphere at frontal sites. Indeed, *CN* amplitude values did not differ as a function of cueing conditions over right frontal sites, as can be seen in Fig. 8.

## Discussion

In this study, according to mere behavioral data the increased alertness induced by central or double cues did not reduce the costs for overcoming the perceptual and motor conflict induced by flankers. Infact, RTs did not differ among the CC, 2C or NC conditions inresponse to incongruent trials. The only benefit, also able to annul target-related conflict was observed for the LC spatially informative attentional condition - no difference in RTs to congruent vs. incongruent trials was found for this cueing condition - greatly enhancing the speed of processing. The latency of Conflict Negativity component of ERPs strongly agreed with response times, by providing the same identical pattern of results: an advantage of LC condition with respect to all other conditions. In this respect, both our behavioral and ERP latency data suggest an integration of orienting and executive control, but not alerting, attention networks, unlike what reported by Fan *et al*.^2^ using different ANT operational scoring procedures. Indeed, in their study these authors advanced a model implying a parallel engagement and completely independent functioning of the executive control, alerting and orienting networks of attention in the brain. This earlier conceptualization of independence among the attention networks, was, however, revised by this same research group, based on the findings that the cost of incongruence was significantly enhanced for situations in which an alerting cue had no spatial value (i.e., in CC and 2C conditions), as compared to situations in which the cue was space-specific or was not delivered at all (i.e., in the LC and NC conditions, respectively), in a later RTs study^8^. These results were interpreted as indicating a functional integration and interaction among these attention networks. RT and *CN* peak latency findings of our study are fully in agreement with Fan’s *et al*.^8^ results and their networks integration interpretation.

Importantly, proving to be more sensitive to the experimental manipulations than behavioral indices, our *CN* measures also showed an increase in *CN* amplitude to targets preceded by a pre-alerting cue (i.e., both CC and 2C) than to altogether previously uncued targets (NC). Unlike RTs, then, our ERP findings suggest an intermediate benefit for conflict detection and resolution induced by a previous phasic increase in alerting state. Still more importantly, our data also indicate that a previous valid orienting of attention-spotlight to target spatial location overcame the interference due to the visual distracters, thus annulling the congruent vs. incongruent difference. These dramatic differences in brain processing of conflicting target-flanker patterns when preceded by a phasic alerting state with respect to when preceded by a voluntary focusing of attention onto their spatial location were revealed only by electrophysiological analyses, which, in comparison to behavioral measures, proved to be less sensitive to them.

An important issue to consider here is that, overall, our findings mesh well with Yeung’s *et al*.^27^ model proposing that, in correct motor response trials, conflict-related ERPs activity, such as the N2 component, would always precede motor responses. Consistent with one of the tenets of this model, our results clearly indicate that, notwithstanding the modulation of *CN* morphological features as a function of the diverse cueing conditions, this electrophysiological activity always preceded RTs latency as N2 component in Yeung’s *et al*.^27^ study. It is conceivable, then, that the different patterns of results obtained with ERPs and RTs may somehow be due to the differing amount of further processing interposed between participants’ bioelectric *CN* activation and related motor response. It might indeed be possible that a top-down, semantic evaluation of the certainty to have correctly identified target nature, as possibly mirrored at the scalp by the development of a later P3a and P3b positive complex, might be required after conflict monitoring and reduction at N2 processing level. Most likely, the processing costs of this semantic evaluation, as reflected in the *CN* activity, may be much lower when attention-spotlight had already previously been focused onto the targets delivery location (as in LC condition), supposedly by making the distinction between the target and flankers more clearly defined and less subject to confusion, than when their processing was preceded by a mere phasic alerting, or, in turn, by none pre-cueing, followed by a reflexive orienting of attention-spotlight to their unpredicted location (as in 2C and CC or NC conditions). As a consequence, it would derive that latency and amplitude features of the *CN* obtained at the scalp should be, in principle, much more alike to N2 in the LC than in the other precueing conditions (See Figs 3 and 4 again), and, as such, more related to ACC than to DLPC activations, in the top-down orienting condition than in all other conflict-monitoring reflexive conditions. In our view, these are relevant findings that should be taken into account by the conflict monitoring model^27^.

Additionally, as much as our RT results were consistent with previous reports demonstrating that the amount of interference generated by flankers was not modulated by the reflexive orienting of spatial attention to targets location^2^,^8^,^36^ in both the ANT pre-cued phasic alerting (i.e., 2C and CC) and uncued tonic-alerting conditions (NC), our ERP-DWs data suggest that, unlike in the former conditions, in the latter one conflict control might have occurred merely on the bases of an unexpected reflexive orienting-related appraisal of target-flankers interference as reflected by *CN*. This might have been due to the fact that uncertainty of target correct discrimination from flankers should have been in this case the highest (see Figs 3 and 4). All in all, it follows, then, that the diversities across the post-targets conflict processing modes envisioned by means of ERPs *CN* as a function of the different attention orienting and alerting functions might be, instead, abated, at motor output levels, for the alerting conditions. Hypothetically, this might be due to the greater degrees of uncertainty associated to target-flankers detection and discrimination, as reflected by a greater amount of later P3 complex activity in the *CN*, in the pre-alerted conditions than in the spatially focused one.

Importantly, the finding of a larger *conflict negativity* amplitude over frontocentral sites for the incongruent condition indicate a central and anterior scalp distribution for this component, which fits very well with the hypothesis of an anterior cingulate^26^,^27^,^28^,^29^,^40^,^41^ and dorsolateral prefrontal generator^42^, advanced in other studies with similar paradigms involving conflict resolution.

For example, Van Veen and Carter^17^ found a larger N2 response to conflicting trials at central sites (Cz) at about 340–380 ms of latency and identified the neural generator of this response in the caudal part of anterior cingulate cortex (cACC). However, in their specific study the N2 might have been affected by a motor-preparation constituent factor that was not present in our own study. Indeed, distracters were letters that indicated the hand to be used for motor response 100 ms prior to target presentation. Therefore, the decision relative to the effector choice occurred before the appearance of target, which might possibly explain the cACC generator for N2, reflecting, among other factors, also a motor constituent factor. Conversely, in our study any motoric explanation of lateralized activations *prior* to target appearance can be ruled out, since no information about the hand of response was provided before the target arrow was presented.

Our data also showed that *Conflict Negativity* was overall larger, and earlier, over right scalp sites independent of cueing conditions, as proved by the analyses performed on peak latency, amplitude and scalp-surface surface isopotentials mapping. Considering that we have found that executive control of target-related conflict was somehow boosted by greater alerting levels, this might be interpreted in the light of the literature according to which there would be a right-hemispheric asymmetry in the cerebral vigilance system. This has been suggested to rely mostly upon a right-hemisphere network, based on noradrenergic projections from the locus coeruleus to the frontal lobes^43^,^44^,^45^ and also involving activations in the parietal cortex^46^. The key role of NA-driven structures, and of the right hemisphere involvement in intrinsic alerting and attention has been confirmed by several neuroimaging studies (e.g., see refs 42, 46 and 47) showing activations in the reticular formation together with the right-sided ACC. In addition, a PET study^48^, involving auditory processing, showed a very similar pattern of activation of the right dorso-lateral prefrontal cortex (DLPFC) and ACC, together with thalamic foci, thus suggesting supra-modality for the right-hemisphere network. Our findings of a right hemispheric asymmetry in *CN* latency and amplitude fully agree with the abovementioned literature. Besides, they are fully consistent with recent behavioral evidence of a left visual field/right-hemispheric advantage for invalid unattended targets found by Spagna *et al*.^9^ using an adapted version of ANT paradigm in which target-flanker patterns fell in the left or right visual hemifields as horizontally lateralized across the vertical meridian. In our view, the full consistence of Spagna’s *et al*.^9^ right-sided asymmetry with our own finding of aright-hemispheric asymmetry, based on the presentation of vertically located targets across the visual horizontal meridian, lends very strong support for the view of anactivation of an alerting-regulating network hardwired to the right hemisphere.

As much as CN was, as a whole, of greater amplitude over right frontocentral scalp areas, however, we found that cueing-related modulation of this ERPs response was lateralized to the left hemispheric sites, especially at frontal electrodes. This suggests the co-presence of multiple generators differently involved in conflict monitoring, and in the endogenous attentional resolution of conflict itself. Consistently, in a very interesting study Huster and coworkers^49^ addressed the issue of hemispheric asymmetry in ACC functionality by observing subjects with a low vs. high degree of left hemispheric midcingulate fissurization while collecting behavioral as well as ERP correlates of Stroop task-related interference. Indeed, although the cingulate cortex seems to be, at first sight, a unitary structure, recent research showed a high degree of functional as well as structural variability. In their study, a high degree of left fissurization (i.e., extension of paracingulate sulcus) was associated with decreased behavioral Stroop interference accompanied by a stronger and prolonged frontal negative potential to incongruent trials starting around 320 ms. This increase in frontal negativity was assumed to reflect an enhanced activity of a conflict monitoring system located in the midcingulate cortex. Overall, their data showed that a more pronounced dissociation of ERPs to congruent and incongruent trials found between 320 and 600 ms in human individuals with a left mid-cingulate asymmetry indicated a differential and likely higher ability in conflict monitoring. These results agree with our findings of a lack of right-sided, frontal pre-cueing-related modulation of the post-target *CN* outcome.

Overall, our data also fit well with a model of phasic and tonic alerting as independent but interacting systems: when a speeded response is required to a target shortly preceded by a warning stimulus, i.e., under phasic alerting conditions, the system relies only partially upon right hemisphere activation. More crucial would be the activation of left hemisphere structures seen to be involved by the abovementioned neuroimaging studies on phasic alerting activations. In line with this pattern of results, an event-related fMRI study by Fan *et al*.^3^ showed stronger cued-alerting activations in the superior and inferior parietal cortices, and in the frontal lobe of the left hemisphere as compared to those of the right hemisphere.

Overall, the spatio-temporal profiles of voltage scalp topographies (see Fig. 7) for the conflict-related neural activity suggests that the medial, including ACC, and dorsolateral prefrontal regions may be involved in attentional allocation and conflict resolution. However, the data deserve further investigation, with the aid of inverse solution source reconstruction techniques, for determining more thoroughly not only brain network responsible for processing dynamics during conflict control and resolution in relation to attention and alerting, but also the specific roles of left and right hemispheric structures in these dynamics.

## Additional Information

**How to cite this article**: Zani, A. and Proverbio, A. M. How voluntary orienting of attention and alerting modulate costs of conflict processing. *Sci. Rep.* **7**, 46701; doi: 10.1038/srep46701 (2017).

**Publisher's note:** Springer Nature remains neutral with regard to jurisdictional claims in published maps and institutional affiliations.
